# Adiponectin levels and expression of adiponectin receptors in isolated monocytes from overweight patients with coronary artery disease

**DOI:** 10.1186/1475-2840-10-14

**Published:** 2011-02-01

**Authors:** Anastasios Kollias, Panayoula C Tsiotra, Ignatios Ikonomidis, Eirini Maratou, Panayota Mitrou, Erifyli Kyriazi, Eleni Boutati, John Lekakis, Theofanis Economopoulos, Dimitrios T Kremastinos, George Dimitriadis, Sotirios A Raptis

**Affiliations:** 1 Hellenic National Center for the Research, Prevention and Treatment of Diabetes Mellitus and its Complications (H.N.D.C), 3 Ploutarchou, 10675 Athens, Greece; 2 2nd Department of Internal Medicine, Research Institute and Diabetes Center, Athens University Medical School, "Attikon" University General Hospital, 1 Rimini, 12462 Haidari, Athens, Greece; 3 2nd Department of Cardiology, Athens University Medical School, "Attikon" University General Hospital, 1 Rimini, 12462 Haidari, Athens, Greece

## Abstract

**Background:**

Adiponectin has insulin-sensitizing and anti-atherosclerotic effects, partly mediated through its action on monocytes. We aimed to determine adiponectin levels and expression of its receptors (AdipoR1 and AdipoR2) in peripheral monocytes from overweight and obese patients with coronary artery disease (CAD).

**Methods:**

Fifty-five overweight/obese patients, suspected for CAD, underwent coronary angiography: 31 were classified as CAD patients (stenosis ≥ 50% in at least one main vessel) and 24 as nonCAD. Quantitative RT-PCR and flow cytometry were used for determining mRNA and protein surface expression of adiponectin receptors in peripheral monocytes. A high sensitivity multiplex assay (xMAP technology) was used for the determination of plasma adiponectin and interleukin-10 (IL-10) secreted levels.

**Results:**

Plasma adiponectin levels were decreased in CAD compared to nonCAD patients (10.9 ± 3.1 vs. 13.8 ± 5.8 μg/ml respectively, p = 0.033). In multivariable analysis, Matsuda index was the sole independent determinant of adiponectin levels. AdipoR1 and AdipoR2 protein levels were decreased in monocytes from CAD compared to nonCAD patients (59.5 ± 24.9 vs. 80 ± 46 and 70.7 ± 39 vs. 95.6 ± 47.8 Mean Fluorescence Intensity Arbitrary Units respectively, p < 0.05). No significant differences were observed concerning the mRNA levels of the adiponectin receptors between CAD and nonCAD patients. AdipoR2 protein levels were positively correlated with plasma adiponectin and Matsuda index (r = 0.36 and 0.31 respectively, p < 0.05 for both). Furthermore, basal as well as adiponectin-induced IL-10 release was reduced in monocyte-derived macrophages from CAD compared to nonCAD subjects.

**Conclusions:**

Overweight patients with CAD compared to those without CAD, had decreased plasma adiponectin levels, as well as decreased surface expression of adiponectin receptors in peripheral monocytes. This fact together with the reduced adiponectin-induced IL-10 secretion from CAD macrophages could explain to a certain extent, an impaired atheroprotective action of adiponectin.

## Background

Cardiovascular disease (CVD) is a leading cause of mortality worldwide accounting for 30% of total global deaths and the number one leading cause of deaths in developing countries. Various studies have demonstrated the association of obesity and insulin resistance with cardiovascular risk. It has been recognized that inflammatory mechanisms play a pivotal role in initiation, maintenance and progress of CVD and that the association of CVD with obesity is mediated through the adipose tissue secretory activities.

Adiponectin, an adipose tissue secreted protein, has been well recognized to exhibit insulin-sensitizing, anti-inflammatory and anti-atherosclerotic properties, which are mediated through its receptors, AdipoR1 and AdipoR2 [1,2]. These receptors are ubiquitously expressed in most organs as well as in human peripheral monocytes, and in monocyte-derived macrophages [2-5]. In these cells, adiponectin has been shown to modulate their inflammatory activity and most importantly to inhibit their transformation to foam cells, a hallmark of atherosclerosis [6-10]. More specifically, adiponectin induces the anti-inflammatory cytokine IL-10 in human monocytes and macrophages [5,6,11], while it suppresses the LPS-stimulated release of IL-6 in porcine macrophages [8]. Furthermore, adiponectin-induced secretion of IL-6 and IL-8 is reduced in monocytes from patients with type 1 or type 2 diabetes [5,12]. This fact may have significant implications in terms of atherosclerotic processes, since these cells play a pivotal role in inflammation and atherosclerosis [13].

Adiponectin levels are known to be decreased in patients with obesity, type 2 diabetes and coronary artery disease (CAD) [14-17]. However, in recent studies, adiponectin was shown to be associated with an adverse outcome in patients with CAD, challenging the emerging evidence for its role [18-21]. In addition, alterations at the level of adiponectin receptors expression have been reported but with inconclusive results showing upregulation or downregulation in the presence of insulin resistance [5,22-28]. Recently, a lower abundance of adiponectin receptors was found in monocytes from overweight/obese patients with type 2 diabetes [5]. However, the expression levels of adiponectin receptors have not been examined in CAD.

The aim of this study was to examine whether the relative mRNA and protein (surface) expression of AdipoR1 and AdipoR2 in human peripheral monocytes is altered in overweight/obese patients with CAD, and whether this might relate to their circulating adiponectin levels and to indices of insulin resistance and atherosclerosis.

## Methods

### Subjects and investigation

Our study included patients with Body Mass Index (BMI) above 25 Kg/m^2 ^who underwent elective coronary angiography for the investigation of the existence of chronic stable CAD. Patients with a 50% or greater diameter stenosis in at least one major coronary artery were considered as CAD positive patients. The exclusion criteria were: unstable angina or acute myocardial infarction, unstable condition including infections, heart failure, malignancies, renal disease (creatinin level > 1.5 mg/dl). All patients underwent intima-media thickness (IMT) assessment in 2 paired segments of both common carotids, as well as in carotid bulbs using B-mode ultrasound imaging. IMT was measured using an automatically trading software (Vivid 7 GE Horton, Norway). The research conducted in this paper has been carried out in accordance with the Declaration of Helsinki (2000) of the World Medical Association and the study protocol was approved by the Scientific and Ethics Committee of "Attikon" University General Hospital. All patients gave their written informed consent.

Two to three weeks after their discharge from the Cardiology Department, patients were subjected to a 2-hour oral glucose (75gr) tolerance test (OGTT) for the assessment of their insulin resistance status. All subjects were informed to halt their medication at least one day before the OGTT, as necessary. Insulin resistance was calculated by the homeostasis model assessment index (HOMA-IR) as [fasting insulin (*μ*U/mL) × fasting glucose (mmol/L)]/22.5 [29]. Insulin sensitivity was calculated by the Matsuda index as [10.000/square root of (fasting glucose × fasting insulin) × (mean glucose during OGTT × mean insulin during OGTT)] [30]. At the beginning of the OGTT, fasting blood was drawn and plasma was kept at -80°C for measurement of adiponectin levels. Fasting triglycerides, total and HDL- cholesterol were determined by an ILAB analyser (Instrumentation Laboratory SpA, Italy). Plasma glucose levels at fasting and during the OGTT, were analyzed on a Falcor 300 Chemical Analyser (Menarini Diagnostics, Italy), while plasma insulin levels were measured by IRMA (INSI-CTK, DiaSorin, Italy), in all individuals.

BMI was calculated as the ratio of body weight (Kg) per height (m^2^). Waist and hip circumference, weight and height were also measured in all individuals. Fifty-five patients, aged between 41 and 75 years, were eligible to be included in the study among 71 subjects screened. All patients were of a stable weight and had been on a normal isocaloric diet with normal physical activity, not participating in any specific exercise program. Information about use of medication, such as antihyperlipidemic, antidiabetic and antihypertensive medication was also collected. None of the patients were taking thiazolidinedione medication.

### Study of adiponectin receptors surface (protein) expression with flow cytometry

**Figure 1:**
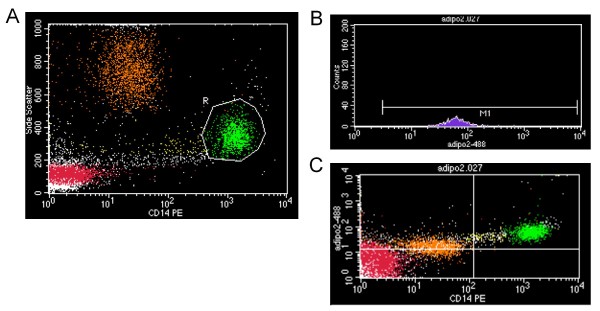
**Flow cytometric analysis of adiponectin receptors in isolated monocytes**. (A) Dot plot presentation of isolated mononuclear cells. Cells subpopulations were distinguished by granularity (side scatter) versus staining with CD14-PE monoclonal antibody. Monocytes (CD14-PE^+^) were gated (R). (B) Analysis of Adipo-R2 log fluorescence of the gated monocytes. (C) Dot plot of the Adipo-R2 expression versus CD14-PE. The upper right quadrant includes the cells showing positive expression for both adipoR2 and CD14 antibodies (green colour).

The mononuclear cell fraction was isolated from peripheral blood and the surface adiponectin receptors, AdipoR1 and AdipoR2, were determined after staining the cells with suitable antibodies. More specifically, the antibodies used were rabbit anti-mouse AdipoR1 and rabbit anti-mouse AdipoR2 (Alpha Diagnostic International, San Antonio, USA), each corresponding to the extracellular carboxy-terminus domain of the respective receptor. Since the antibodies were not fluorochrome conjugated, they were labelled with the Zenon™ Alexa Fluor^® ^488 Rabbit IgG labelling kit (Invitrogen, Carlsbad, CA, USA). Cells were incubated for 30 min with Alexa Fluor^® ^488-conjugated immunoglobulin in a ratio 1 × 10^6 ^cells μg^-1 ^of immunoglobulin under mild constant shaking. The monocyte fraction was simultaneously stained with anti-CD14-PE monoclonal antibody (BD Biosciences, San Jose, CA, USA). After incubation with the antisera, cells were fixed with 0.1% (w/v) paraformaldehyde. A histogram of log green fluorescence of each adiponectin receptor was used for the determination of the mean fluorescence intensity of each sample (Figure 1).

The specificity of the antibodies used was evaluated by staining cells with isotype controls suitable for each antisera and the blockage of Fc-receptors prior to staining [31].

Two-colour flow cytometric analysis was performed on a BD FACSCalibur 4 colour flow cytometer (BD Biosciences) as previously described [31]. Data acquisition and analysis were performed using the BD CELL Quest Pro software (BD Biosciences). Results are expressed in mean fluorescence intensity arbitrary units (MFI AU).

### Isolation of peripheral monocytes

Monocytes were isolated from mononuclear cells using a Magnetic Cell-Sorting technique with CD14 Human MicroBeads (Miltenyi Biotec, Gladbach, Germany). The exact number of cells was determined cytometrically with Flow-Count™ Flurospheres (Beckman-Coulter, Miami, USA). The purity of the magnetically isolated CD14^+ ^monocytes was determined after staining of the cells with a CD14 PE-CD45 FITC antibody (BD Biosciences, USA)

### RNA preparation

Total RNA was extracted from CD14^+ ^monocytes using the Tripure Isolation Reagent (Roche Diagnostics GmbH, Mannheim, Germany), and the integrity of RNA samples was determined on ethidium bromide-agarose gels and spectrophotometrically. For the removal of any residual DNA contamination, RNA samples were pre-incubated with RQ1 RNase-free DNase (Promega, Madison, WI, USA) before the reverse transcription reaction. Reverse transcription of 1 μg of total RNA was performed in all samples with the Transcriptor First Strand cDNA Synthesis Kit using random hexamer primers, according to the manufacturer's instructions (Roche Diagnostics GmbH, Mannheim, Germany).

### Relative real-time PCR quantification of adiponectin receptors mRNA expression

A quantitative real-time PCR using fluorescently labeled hybridization probes was developed for detecting relative AdipoR1 and AdipoR2 mRNA levels in human CD14^+ ^monocytes, in a spectrofluorometric thermal cycler (LightCycler, ROCHE, Manheim, Germany) as previously described [32].

Hybridization specific primers and fluorescently labelled hybridization specific probes for all genes were designed and manufactured by the TIM-MOLBIOL (Berlin, Germany). Primers were chosen to lie between exons whenever possible and the analysis was performed with the program OLIGO 6.0 (Molecular Biology Insights, Cascade, USA). The specific reaction conditions for each set of genes (primer concentration, annealing temperature, magnesium chloride) were optimized using first the SYBR Green fluorescent dye (LightCycler^® ^FastStart DNA Master SYBR Green I, Roche Diagnostics). Hybridization primers (sense and a-sense, respectively) for the targets (AdipoR1, AdipoR2) and reference genes (β-actin) were as follows: for AdipoR1: 5'-TTTGACATATGGTTCCAGTCTCA-3' and 5'- GACTCTTCCTCTCACTTCAGCAA-3', for AdipoR2: 5'-TGACATCTGGTTTCACTCTCATCAG-3' and 5'-GTCATAGTCCCTGGAGACTGGT-3', for β-actin: 5'-CTTCTACAATGAGCTGCGTGTG-3' and 5'-GTGAGGATCTTCATGAGGTAGTCAGTC-3'. Hybridization probes (FL- and LC Red640-probes, respectively) for the targets (AdipoR1, AdipoR2) and reference genes (β-actin) were as follows: for AdipoR1: 5'-CCGTAACGGAATTCCTGAAGGTTGGAGAC-3' and 5'-CCATAGAAGTGGACAAAGGCTGCTGCC-3', for AdipoR2: 5'-CCTGGAGGTTTGAGACACCATGGAAG-3' and 5'-GAACAAAAGCTCCAGCAACCACAAAGATA-3', for β-actin: 5'- GGTATGCCCTCCCCCATGCC-3' and 5'- TCCTGCGTCTGGACCTGGCTG-3'.

Relative quantification is the method that determines the changes in steady-state mRNA levels of a gene across various samples and expresses them relative to the levels of an internal reference control gene, usually a housekeeping gene. Standard curves describing the PCR efficiencies of the target (AdipoR1 and AdipoR2) and the reference genes (β-actin) were created from a dilution series of the calibrator cDNA (HL60 cDNA induced with phorbol myristyl acetate -PMA-), using the Second Derivative Maximum Method with the Arithmetic baseline adjustment for the determination of the various crossing points. To quantify the relative expression levels of Adipo-Rs mRNA in the various samples, we run the target and the reference gene of each sample, along with the calibrator cDNA, and the final results were calculated using the LightCycler Relative Quantification 1.0.1 Software (ROCHE, Manheim, Germany). Samples were run in duplicates or triplicates and results are expressed in arbitrary units (AU). The results from different PCR runs were run randomly in ethidium bromide stained agarose gels and photographed in a GelDoc-It 300 Imaging System (UVP, Cambridge, UK).

### Cell culture

Mononuclear cells from peripheral blood were resuspended in RPMI-1640 medium (GIBCO-INVITROGEN Corp, Carlsbad, CA, USA), supplemented with 10% human type AB male serum (SIGMA-ALDRICH Corp, St. Louis, MO, USA) and 1% Penicillin/streptomycin (5000 IU/ml-5000 μgr/ml) (GIBCO-INVITROGEN Corp, Carlsbad, CA, U.S.A) and incubated in 10 cm cell culture dishes for 1 hour. Non-adherent cells were removed and the remaining adherent cells were collected, counted and aliquoted in a 24-well plate at a density of 2-3 × 10^6 ^cells/well. Cells were left in a 37°C CO_2 _incubator for 7 days and the medium was changed every two days. After that period, human monocyte-derived macrophages were incubated with human recombinant full-length adiponectin (30 μg/ml) (R&D Systems, Minneapolis, MN, USA) for further 48 hours. At the end of the incubation period, cell supernatants were collected, aliquoted and kept at -80°C for measurement of released IL-10.

### Immunoassays

Circulating levels of adiponectin were measured in the plasma of all subjects, using a high sensitivity multiplex assay (xMAP technology) and the fluorescently labeled microsphere beads (HCVD1-67AK Lincoplex kit, Millipore Corp., MA, USA), in a LUMINEX 200 instrument (Luminex Corp., USA). Sensitivity of the assay was 56.0 pg/ml, intra-assay and inter-assay coefficients of variation were 9.2% and 15.9%, respectively. All samples were diluted 1:100 and measured in duplicate. Samples that could not be detected, were measured again using a higher dilution.

IL-10 secreted levels were measured in the supernatants from human monocyte-derived macrophages using a MILLIPLEX MAP kit (MPXHCYTO-60 K, Millipore Corp., MA, USA), in a LUMINEX 200 instrument (Luminex Corp., USA). Sensitivity of the assay was 0.5 pg/ml and intra-assay and inter-assay coefficients of variation were 5.2% and 9.5%.

### Statistical analysis

Statistical analysis was performed using the SPSS version 14.0.1 software (Chicago, Ill, USA). Data are expressed as mean ± SD. Variables that were not normally distributed (AdipoR1 and AdipoR2 mRNA levels, adiponectin, HOMA index) were logarithmically transformed. Chi square was implemented for categorical variables. Parametric statistical analyses (Pearson's correlation test, unpaired or paired t-test) were applied where appropriate. Multivariate regression analysis (results as mean ± SE), as well as analysis of covariance (ANCOVA) were also performed if any adjustments were necessary. A p-value of less than 0.05 was considered statistically significant.

## Results

### Patients' characteristics

The clinical and metabolic characteristics, as well as the therapeutic regimen of the patients included in the study are presented in Table 1. From the 55 subjects, 46 were males and no differences were observed in BMI between males and females (28.9 ± 2.6 vs. 28.2 ± 2.1 respectively, p = 0.44). Overweight/obese patients with CAD were more likely to be males, while no differences were observed with regard to age, BMI, lipid profile, systolic and diastolic blood pressure compared to similar weight patients without CAD. Patients with CAD had higher values of waist to hip ratio, higher carotid IMT values, decreased levels of plasma adiponectin and decreased insulin sensitivity as assessed by Matsuda index (Table 1). Moreover, the decrease in plasma adiponectin levels remained significant after adjustment for gender (p = 0.046) and BMI (p = 0.025).

**Table 1:** Characteristics of the study population.

	Patients without CAD (n = 24)	Patients with CAD (n = 31)	p value
***Anthropometric characteristics***			
Males (%)	16 (67)	30 (97)	**0.009**
Age (years)	57.6 ± 7.7	60.2 ± 8.5	0.249
BMI (Kg/m^2^)	28.5 ± 2.5	29.1 ± 2.6	0.340
Waist to hip ratio	0.93 ± 0.06	1 ± 0.06	**<0.001**
***Metabolic characteristics***			
LDL-cholesterol (mmol/L)	3.41 ± 0.88	3.12 ± 0.72	0.260
HDL-cholesterol (mmol/L)	1.27 ± 0.28	1.11 ± 0.25	0.078
Triglycerides (mmol/L)	1.31 ± 0.45	1.59 ± 0.15	0.231
Systolic BP (mmHg)	123.5 ± 16.4	128.4 ± 12.3	0.228
Diastolic BP (mmHg)	76.5 ± 9.8	79.6 ± 6.8	0.176
Adiponectin (μg/ml)	13.8 ± 5.8	10.9 ± 3.1	0.033*
***Indices***			
HOMA index	3.52 ± 1.84	5.17 ± 4.78	0.102*
Matsuda index	2.90 ± 1.14	2.28 ± 1.0	**0.041**
Common carotid IMT (mm)	0.86 ± 0.25	1.10 ± 0.30	**0.004**
Carotid bulb IMT (mm)	1.14 ± 0.75	1.49 ± 0.48	0.062
***Drug use***			
Statins (%)	9 (38)	25 (81)	**0.003**
Antiplatelets (%)	8 (33)	27 (87)	**<0.001**
ACE inhibitor, AR antagonists (%)	9 (38)	25 (81)	**0.003**
Nitrates (%)	3 (13)	13 (42)	**0.037**
β-blockers (%)	5 (21)	21 (68)	**0.001**
Calcium channel blockers (%)	3 (13)	7 (23)	0.543
Sulfonylureas (%)	0 (0)	4 (13)	0.192
Metformin (%)	1 (4)	5 (16)	0.329

BMI: Body Mass Index; LDL: Low density lipoprotein; HDL: High density lipoprotein; BP: Blood Pressure; IMT: Intima media thickness; ACE: Angiotensin Converting Enzyme; AR: Angiotensin Receptor

* comparisons in logarithmic scale

### Plasma adiponectin levels and associations

Circulating plasma adiponectin levels were positively associated with Matsuda index and HDL-cholesterol levels, overall (r = 0.315, p = 0.022 and r = 0.29 p = 0.048, respectively, Table 2). In stepwise multivariable analysis with age, gender, waist to hip ratio, the presence of CAD, Matsuda index and the various medications as independent variables, insulin sensitivity (Matsuda index) (beta ± SE: 0.43 ± 0.02, p = 0.024) was the sole independent determinant of adiponectin levels (R^2 ^= 0.10).

**Table 2:** Bivariate correlations of adiponectin and its receptors with the studied variables.

	AdipoR1 protein	AdipoR2 protein	logAdipoR1 mRNA	logAdipoR2 mRNA	logAdiponectin
AdipoR2 protein	0.757**				
logAdipoR1 mRNA	-0.141	-0.258			
logAdipoR2 mRNA	0.003	0.039	0.52**		
logAdiponectin	0.245	0.359**	-0.156	-0.080	
Age (years)	-0.079	-0.081	0.225	0.236	0.098
LDL-C (mmol/L)	0.045	0.025	-0.063	-0.307	0.058
HDL-C (mmol/L)	0.269	0.153	0.052	0.025	0.29*
Triglycerides (mmol/L)	-0.179	-0.145	0.131	0.325*	-0.133
Systolic BP (mmHg)	-0.132	-0.010	0.068	0.303*	-0.006
Diastolic BP (mmHg)	-0.061	0.040	-0.028	-0.018	-0.124
logHOMA index	-0.140	-0.263	0.270	0.146	-0.152
Matsuda index	0.221	0.310*	**-0.342**	-0.035	0.315*
Common carotid IMT (mm)	-0.162	-0.124	0.103	0.189	0.075
Carotid bulb IMT (mm)	-0.082	0.02	-0.123	0.254	0.122

LDL: Low density lipoprotein; HDL: High density lipoprotein; BP: Blood Pressure; IMT: Intima media thickness * p < 0.05, ** p < 0.01

### Expression of adiponectin receptors

**Figure 2:**
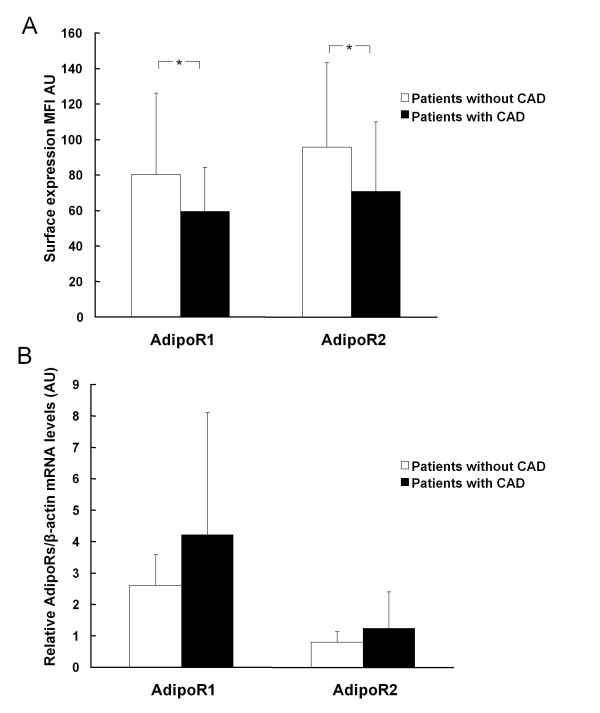
**AdipoR1 and AdipoR2 (A) protein surface and (B) mRNA levels from peripheral monocytes from patients with or without CAD**. * p < 0.05.

Both AdipoR1 and AdipoR2 were found to be expressed in peripheral monocytes at mRNA and protein surface level. While AdipoR1 mRNA was more abundant compared with AdipoR2 mRNA in monocytes (3.5 ± 3.2 vs. 1 ± 0.9 AU, p < 0.001), the opposite was true for the protein surface expression levels (68.2 ± 36.5 vs. 81.3 ± 44.3 MFI AU respectively, p = 0.002). Overweight/obese patients with CAD, apart from the decreased plasma adiponectin levels, had decreased AdipoR1 and AdipoR2 protein surface levels compared to patients without CAD, while they did not show any difference with respect to the levels of AdipoR1 and AdipoR2 mRNA (Figure 2A and 2B). The difference in protein surface expression of AdipoR1 and AdipoR2 with respect to the presence of CAD remained significant for AdipoR1 after adjustment for gender difference (p = 0.033) but was attenuated for AdipoR2 (p = 0.074). However, after adjustment for Matsuda index the difference in protein surface expression was attenuated for both AdipoR1 and AdipoR2 (p = 0.13 and 0.08 respectively).

**Figure 3:**
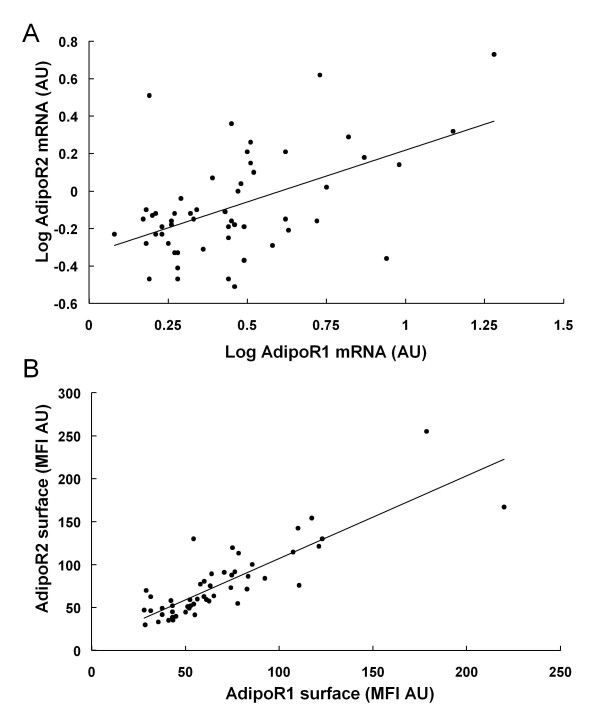
**Correlation between AdipoR1 and AdipoR2 (A) mRNA (r = 0.52, p < 0.001) and (B) surface expression levels (r = 0.757, p < 0.001)**.

**Figure 4:**
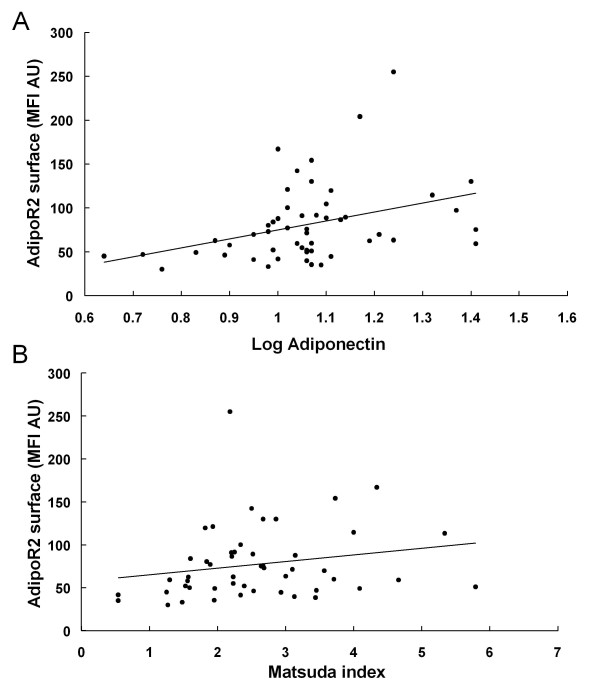
**Correlation of AdipoR2 surface expression levels with (A) plasma adiponectin (r = 0.359, p = 0.009), as well as with (B) Matsuda index (r = 0.31, p = 0.029)**.

Moreover, a robust positive correlation was observed between AdipoR1 and AdipoR2 mRNA, as well as between AdipoR1 and AdipoR2 surface levels (Figure 3). AdipoR1 mRNA levels were inversely correlated with Matsuda index, whereas AdipoR2 mRNA levels were correlated with systolic blood pressure and triglycerides (Table 2). AdipoR2 protein surface levels were associated with plasma adiponectin levels and Matsuda index (Figure 4) and retained this association with plasma adiponectin levels (beta ± SE: 107.58 ± 40.7, p = 0.011) and Matsuda index (beta ± SE: 13.21 ± 6.46, p = 0.047) in multiple regression analysis independent of age, gender and waist to hip ratio. When the latter analysis was confined to the subgroup of CAD patients, AdipoR2 protein surface levels were also correlated with plasma adiponectin levels (beta ± SE: 110.02 ± 48.89, p = 0.034) and Matsuda index (beta ± SE: 25.603 ± 6.060, p < 0.001) independent of age, gender and waist to hip ratio.

Although no associations were revealed overall, between common carotid or bulb IMT with adiponectin receptors mRNA or protein surface levels (Table 2), in subgroup analyses, a positive correlation was found between AdipoR2 protein levels and carotid bulb IMT in CAD patients (r = 0.428, p = 0.026).

### Adiponectin-induced Interleukin-10 production

**Figure 5:**
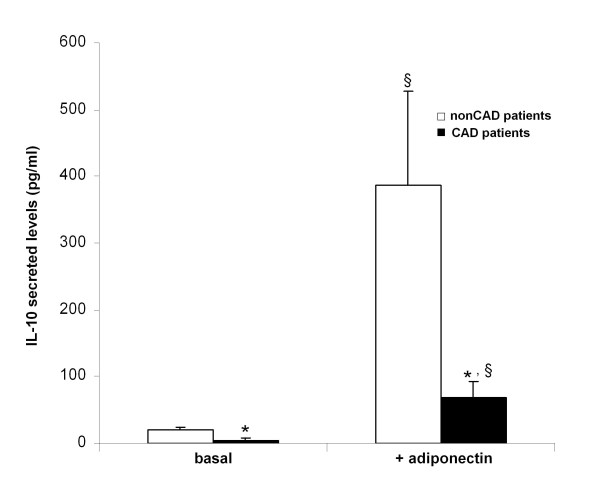
**Basal and adiponectin-induced IL-10 secretion from human monocyte-derived macrophages**. Monocyte-derived macrophages from ten overweight/obese CAD patients and seven nonCAD individuals of similar weight were incubated with human recombinant adiponectin (+adiponectin) or not (basal). *, p < 0.05 vs. nonCAD; §, p < 0.03 vs. corresponding basal levels. Data are expressed as mean ± SE.

In monocyte-derived macrophages from overweight/obese CAD patients, basal IL-10 secreted levels were decreased compared to nonCAD subjects (5.0 ± 7.3 pg/ml vs. 20.3 ± 12.2 pg/ml, p = 0.002) (Figure 5). Moreover, adiponectin treatment of CAD and nonCAD cells significantly induced IL-10 (13.8-fold, p = 0.005 and 19-fold, p < 0.001, respectively), although adiponectin-induced IL-10 release was significantly lower in CAD patients compared to nonCAD subjects (69.2 ± 80.1 pg/ml vs. 386.3 ± 488.2 pg/ml, p = 0.047) (Figure 5).

## Discussion

### Plasma adiponectin levels and associations

Adiponectin is an adipocyte derived protein that exerts anti-inflammatory and anti-atherogenic effects in part by modulating monocyte and macrophage functions. More specifically, adiponectin may prime human monocytes toward the anti-inflammatory M2 phenotype, inhibit the secretion of inflammatory cytokines from monocyte-derived macrophages, prevent monocyte adhesion to endothelial cells and suppress macrophage-to-foam cell transformation [6-10]. In addition, the new adiponectin paralogous protein CTRP-3 has been reported to play a significant role by inhibiting LPS-induced IL-6 and TNF release from human monocytes [33]. Adiponectin receptors have been detected in human monocytes and macrophages [3,4] and recently it was found that variants of the AdipoR2 could be a determinant for atherosclerosis independent of insulin resistance [34]. However the significance of the adiponectin receptors in mediating its beneficial effects in states of CAD has not been examined.

In this study, adiponectin levels were found to be marginally decreased in overweight patients with CAD compared to similar weight control individuals. Although this finding could be expected according to the evidence from previous studies [14], this view is questioned. A couple of studies failed to reveal a relationship of adiponectin levels with early [35] or advanced atherosclerosis [36]. In other studies, adiponectin levels were found to be associated with an adverse outcome in patients with CAD [18-21]. In addition, Rizza et al. reported that adiponectin levels in patients with CAD were not correlated with the severity of CAD, but inversely associated with defects of glucose metabolism [37]. Interestingly, in our study, when adjusting for several factors, adiponectin levels did not differ with respect to the presence of CAD, but were independently associated with Matsuda index. Furthermore, the positive association of plasma adiponectin with HDL-C levels is in agreement with previous epidemiological reports [38,39] and more recent ones suggesting that the apparent correlation of adiponectin with HDL-C is at least partly mediated by a mechanism whereby adiponectin increases the cholesterol efflux from macrophages and thus preventing atherosclerosis [40].

### Expression of adiponectin receptors and adiponectin effects

We confirmed the presence of AdipoR1 and AdipoR2 at both mRNA and protein surface level in peripheral monocytes from subjects with BMI above normal. The robust correlation between mRNA or surface expression levels of the two receptors found, could indicate a synchronous regulation of both genes at transcriptional and translational level. It should be reported that a high correlation between these two receptors at mRNA level has been previously reported in peripheral monocytes from subjects with type 2 diabetes and normal controls, as well as in human skeletal muscle cells [5,26].

However, mRNA and surface expression levels did not exhibit a similar pattern of expression judging from their absolute values and their correlations. In fact, AdipoR1 mRNA levels were higher compared with AdipoR2 respective ones, while the opposite was true for the surface expression levels. In addition, AdipoR1 mRNA levels were inversely correlated with Matsuda index and AdipoR2 mRNA were positively correlated with systolic blood pressure and triglycerides, while surface expression levels of AdipoR2 were positively correlated with Matsuda index and adiponectin levels. Moreover, the presence of CAD was associated with decreased surface expression of both receptors. Although adiponectin receptors are expressed in cells within atherosclerotic carotid plaques [3], we did not uncover any association between adiponectin receptors and carotid IMT values. Yet, in CAD patients, AdipoR2 surface expression levels were correlated with carotid bulb IMT. This finding could represent a compensatory mechanism to decreased adiponectin plasma levels.

The aforementioned results point out that the translation and the transcription process of the adiponectin receptors may not follow the same direction in states of insulin resistance and atherosclerosis. The decreased surface expression levels in combination with the decreased adiponectin levels in CAD could contribute to a vicious circle of decreased adiponectin action and aggravated atherosclerotic processes.

One of the anti-inflammatory actions of the adiponectin is the induction of IL-10 [6]. In our *in vitro *experiments, IL-10 basal protein production was lower in monocyte-derived macrophages from CAD patients compared to that of nonCAD. This fact could be partly attributed to the constant exposure to lower adiponectin plasma levels and to basal reduced AdipoR1 and AdipoR2 surface levels in these cells from CAD patients. Moreover, adiponectin-induced IL-10 secretion was higher in macrophages from nonCAD compared to CAD subjects, although the effect of adiponectin was significant in both CAD and nonCAD cells.

Expression of human adiponectin receptors has been examined mainly at mRNA level only by a limited number of studies which have yielded inconclusive results. Chinetti et al reported that the expression level of AdipoR1 in monocytes decreases during their differentiation into macrophages but remains higher than that of AdipoR2 [3]. However, only AdipoR2 was upregulated by PPAR (peroxisome proliferators-activated receptors) agonists which have insulin-sensitizing properties [3]. These results are in line with our findings, since we also reported a higher level of AdipoR1 mRNA in human monocytes and we found a positive correlation of AdipoR2 surface expression levels with insulin sensitivity. Moreover, protein levels of both adiponectin receptors in monocytes from overweight/obese diabetic patients were reported to be decreased compared to normal weight control subjects [5]. Expression of mRNA levels of adiponectin receptors in human adipose tissue, in states of insulin resistance, has been reported to be either decreased [23-25] or increased [27]. A similar inconsistency has been reported for muscle, where mRNA levels are positively associated with insulin sensitivity [28] or with insulin resistance [22].

### Study limitations

Our study results should be interpreted with regards to some limitations. Firstly, we did not include a control group of normal weight subjects. Nevertheless, this does not invalidate our findings, since the aim of the present study was to examine adiponectin receptors with regard only to the presence of CAD and to correlate their expression with indices of insulin resistance and atherosclerosis. The later became feasible, as our subjects did not differ in terms of BMI values but exhibited a satisfactory variability in insulin sensitivity status, allowing various correlations to be performed. However, it should be noted that CAD patients presented higher values of waist to hip ratio compared to nonCAD patients, suggesting an increased prevalence of visceral adiposity which in turn is a main determinant of adiponectin levels and insulin sensitivity status. In addition, central obesity is associated with increased levels of saturated nonesterified fatty acids, which in combination with hyperinsulinemia, may activate human monocytes to produce proinflammatory cytokines and support the development and propagation of the subacute, chronic inflammatory state [41]. Interestingly, when adjusting for Matsuda index, the difference in the surface expression levels of the adiponectin receptors between CAD and nonCAD patients was attenuated. Moreover, AdipoR2 surface levels in the subgroup of CAD patients were correlated with adiponectin, suggesting that the decrease in AdipoR2 levels in monocytes from CAD patients may be in relation to the decrease in insulin sensitivity. Another limitation of this study is that our patients were not subjected to a satisfactory wash-out period concerning their therapeutic regimen. However, treatment was interrupted at least 24 hours before the measurements and adjustments for drug categories were taken into account in our analyses. Lastly, a limitation of our study could be the relatively small population studied; this could imply a reduced statistical power.

## Conclusions

In summary our data provide evidence that mRNA and surface expression of the adiponectin receptors in human monocytes seems to be subjected to different regulatory mechanisms in states of insulin resistance and atherosclerosis. Furthermore, the latter states are associated with lower adiponectin plasma levels and a decrease in surface expression of adiponectin receptors in monocytes. This, in turn could aggravate the anti-atherogenic action of adiponectin in CAD patients.

## List of abbreviations

AdipoR1: adiponectin receptor 1; AdipoR2: adiponectin receptor 2; AU: arbitrary units; BMI: body mass index; CAD: coronary artery disease; CVD: cardiovascular disease; HDL: high-density lipoprotein; HOMA-IR: homeostasis model assessment index; IL: interleukin; IMT: intima-media thickness; LPS: lipopolysaccharide; MFI AU: mean fluorescence intensity arbitrary units; OGTT: oral glucose tolerance test; RT-PCR: real time-polymerase chain reaction.

## Competing interests

The authors declare that they have no competing interests.

## Authors' contributions

AK: participated in data acquisition, interpretation of data, performed the statistical analysis and drafted the manuscript. PCT: carried out the molecular expression studies, coordinated the study, performed the statistical analysis and drafted the manuscript. II: participated in ultrasound studies and manuscript editing, EM: carried out the immunofluorescence studies, PM and EK: participated in data acquisition, EB: contributed to manuscript editing, JL, TE: conceived and designed the study, DTK, GD, and SAR: revised and gave the final approval of the manuscript. All authors read and approved the final manuscript.
